# Nutrient intakes and top food categories contributing to intakes of energy and nutrients-of-concern consumed by Canadian adults that would require a ‘high-in’ front-of-pack symbol according to Canadian labelling regulations

**DOI:** 10.1371/journal.pone.0285095

**Published:** 2023-05-18

**Authors:** Jennifer J. Lee, Mavra Ahmed, Alena (Praneet) Ng, Christine Mulligan, Nadia Flexner, Mary R. L’Abbé

**Affiliations:** 1 Department of Nutritional Sciences, Temerty Faculty of Medicine, University of Toronto, Toronto, Ontario, Canada; 2 Joannah & Brian Lawson Centre for Child Nutrition, University of Toronto, Toronto, Ontario, Canada; King Faisal University, SAUDI ARABIA

## Abstract

Canada recently mandated front-of-pack (FOP) labelling regulations, where foods meeting and/or exceeding recommended thresholds for nutrients-of-concern (i.e., saturated fat, sodium, and sugars) must display a ‘high-in’ FOP nutrition symbol. However, there is limited research on the amounts and sources of foods consumed by Canadians that would require a FOP symbol. The objective was to examine the intakes of nutrients-of-concern from foods that would display a FOP symbol and to identify the top food categories contributing to intakes for each nutrient-of-concern. Using the first day 24-hour dietary recall from the nationally representative 2015 Canadian Community Health Survey-Nutrition (CCHS), Canadian adults’ intakes of nutrients-of-concern from foods that would display a FOP symbol was examined. Foods were assigned to 1 of 62 categories to identify the top food categories contributing to intakes of energy and nutrient-of-concern that would display a FOP symbol for each nutrient-of-concern. Canadian adults (n = 13,495) consumed approximately 24% of total calories from foods that would display a FOP symbol. Foods that would display a FOP symbol for exceeding thresholds for nutrients-of-concern accounted for 16% of saturated fat, 30% of sodium, 25% of total sugar, and 39% of free sugar intakes among Canadian adults. The top food category contributing intakes of each nutrient-of-concern that would display a FOP symbol were nutrient-specific: Processed meat and meat substitutes for saturated fat; Breads for sodium; and Fruit juices & drinks for total and free sugars. Our findings show that Canadian FOP labelling regulations have the potential to influence the intakes of nutrients-of-concern for Canadian adults. Using the findings as baseline data, future studies are warranted to evaluate the impact of FOP labelling regulations.

## Introduction

Poor diet is one of the major preventable risk factors for non-communicable diseases (NCD) [1, 2]. Mandatory government regulations and government-led voluntary recommendations have been introduced in many countries to endorse the use of front-of-pack (FOP) labelling to support healthy dietary intakes of the population [3–6]. FOP labelling refers to the use of a simple and easy-to-understand symbol displayed on the front of food and beverage (‘foods’, hereafter) packages to communicate the healthfulness of the food [4, 7]. FOP labelling has been shown to improve the diet quality of a population both by influencing individual dietary behaviours at the point-of-purchase [8, 9] and by improving the nutritional quality of the food supply system through manufacturer-driven product reformulations [10, 11]. Experimental studies using FOP labels have been shown to decrease consumer purchasing intentions for foods displaying FOP labels for nutrients-of-concern [12, 13]. In Chile, following the implementation of mandatory FOP labelling regulations, household purchases of beverages displaying a ‘high-in’ FOP label decreased by over 20% compared to expected purchases based on pre-regulations purchasing trends [13]. Further, there was an overall decrease in the proportion of products displaying ‘high in’ FOP labels for energy, saturated fat, sugar, and/or sodium (51% vs. 44%) in the Chilean food supply following the implementation of the FOP labelling regulations [14]. In July 2022, final Canadian FOP labelling regulations were published, mandating pre-packaged foods meeting and/or exceeding threshold levels of nutrients-of-concern (i.e., saturated fat, sodium, and sugars) to display a ‘high-in’ FOP nutrition symbol (‘FOP symbol’ hereafter) as of January 1, 2026 [15].

Previous studies have shown that Canadians exceed intakes of nutrients-of-concern. Canadians, on average, consume 10.4% of total energy intake from saturated fat [16] (recommended levels <10% of total energy intake [17]), 2,760 mg/d of sodium [18] (Chronic Disease Risk Reduction [CDRR] level <2,300 mg/d [19]), and 13.3% of total energy intake from free sugars [20] (recommended level <10% of total energy intake [21]). Modelling studies have shown that food reformulations [22] and behavior changes [23, 24] have the potential to decrease the consumption of nutrients-of-concern, including saturated fat, sodium, and/or sugars, and reduce the risk of diet-related NCDs. Although numerous countries have introduced or implemented mandatory FOP labelling regulations [6, 25] and current evidence around the world [8, 9] have shown that FOP labelling has the potential to improve purchasing and consumption behaviors, there is limited data on monitoring and evaluation of the regulations, likely due to the recent introduction/implementation of such regulations. To date, only two studies from Chile, which mandated FOP labelling regulations in 2016, examined the impact of FOP labelling regulations by comparing differences in household purchases of ‘high in’ labelled beverages [13] and nutrients-of-concern [26] before and after the policy implementation. Data on the amounts and contributing food categories consumed that would require a FOP symbol, particularly pre- and post-policy implementation, is lacking, and such data is needed to evaluate the impact of regulations and make improvements to achieve policy objectives. Therefore, the objectives of this study were: (i) to estimate the intakes of energy and nutrients-of-concern from foods consumed by Canadian adults that would be required to display a FOP symbol under the Canadian FOP labelling regulations; and (ii) to identify the top food categories contributing to energy and nutrient-of-concern intakes from foods that would display a FOP symbol. These data will provide a useful baseline to evaluate changes once the regulation come in force in January 2026.

## Materials and methods

### Canadian Community Health Survey—Nutrition 2015

Data from the 2015 Canadian Community Health Survey (CCHS)–Nutrition Public Use Microdata File were used in this study [27]. CCHS-Nutrition is a nationally representative, cross-sectional survey with data from 20,487 Canadians, conducted by Statistics Canada in 2015 [28]. CCHS consists of two surveys per household: 1) a 24-hour dietary recall to assess all foods consumed by an individual over 24 hours, and 2) a general health questionnaire to collect self-reported sociodemographic, anthropometric, and health data. CCHS includes data from all individuals >1 y living in private dwellings in the 10 Canadian provinces, excluding full-time members of the Canadian Forces or those who live in the Territories, on reserves and other Indigenous settlements, in some remote areas, or institutions (e.g., prisons or care facilities) [28]. A subset of CCHS respondents was invited to complete a second 24-hour recall by phone 3–10 days following the initial survey; however, in this study, only the first 24-hour dietary recall data from adults (≥19 y) was used for the analysis. Out of 20,487 respondents in the CCHS, data from respondents who were below 19 years of age (n = 6,568), underweight (Body Mass Index [BMI] <18.5 kg/m^2^; n = 230), lactating (n = 183), or did not report any food consumption (n = 11) were excluded from the analysis. The final analytic sample consisted of 13,495 respondents.

Misreporters of energy intake were identified using the ratio of their reported Energy Intake (EI) to their estimated Total Energy Expenditure (TEE) for each respondent, as previously reported [29–31]. Briefly, TEE was calculated based on age, sex, BMI, and physical activity levels (i.e., sedentary, low active, moderately active, and highly active) using the Institute of Medicine equations [32]. For data from respondents without measured height and weight, self-reported height and weight were used after adjusting the values using a Statistics Canada correction factor [29, 33]. Respondents’ average physical activity per day in minutes was calculated using the reported hours of physical activity per week, and physical activity levels were categorized into sedentary, low active, active, and very active as per common Health Canada methodology [28]. For respondents that did not disclose any anthropometric information, estimated calorie requirements by age, sex, and physical activity levels in the Dietary Guidelines for Americans 2020–2025 [34] were used to estimate TEE. Under- and over-reporters were defined as respondents with the ratio of EI:TEE *<*0.7 and *>*1.42, respectively, while plausible reporters were defined as respondents with EI:TEE ratio of 0.7–1.42 [30].

### Canadian Nutrient File database

Foods reported in CCHS were matched to the Canadian Nutrient File (CNF) database created by Health Canada to obtain nutrient intakes of all reported foods. CNF is a generic food composition database of 6,904 commonly-consumed fresh, pre-packaged, and prepared foods with over 150 nutrients [35]. The nutrient information in CNF is derived from the United States Department of Agriculture (USDA) National Nutrient Database for Standard Reference with modifications for Canadian levels of fortification and regulatory standards, as necessary; Canadian-specific foods; and other Canadian data from some brand name foods and commodities.

All foods in CNF were categorized by Health Canada’s Table of References Amounts for Food (TRA), which represents the amount of food typically consumed in one sitting and serves as the basis for determining serving sizes (i.e., reference amount) in the Nutrition Facts table (NFt) [36]. Health Canada’s TRA categories consist of 24 major and 188 minor categories. To identify top food categories contributing to intakes of energy and each target nutrient-of-concern, similar TRA minor categories were grouped together (e.g., light-, medium-, and heavy-weight cakes, coffee cakes, brownies, muffins, and cookies combined under “Cakes, cookies, and other baked goods”) to create a modified list of 62 food categories (**S1 Table**). The modified categories were used to limit similar foods from being underrepresented when analyzed at a population level.

### Canadian front-of-pack labelling regulations

Using the details of FOP labelling regulations published in *Canada Gazette II* [15], a nutrient profiling model was developed to classify all foods in CNF. Briefly, Canadian FOP labelling regulations mandate that pre-packaged foods display a FOP symbol for meeting and/or exceeding threshold levels for 3 target nutrients-of-concern: saturated fat, sodium, and/or total sugars. **Table 1** shows the thresholds (%DV and absolute amount per nutrient) used to identify foods ‘high in’ nutrients-of-concern according to Canadian FOP labelling regulations. The thresholds are set based on the percent Daily Value (%DV) per reference amount for each nutrient based on the age groups and the reference amount, resulting in 6 different thresholds: (i) 10% DV for foods for adults and children >4 years of age with a reference amount ≤30 g or 30 mL; (ii) 15% DV for foods for adults and children >4 years of age with a reference amount >30 g or 30 mL; (iii) 30% DV for foods for adults and children >4 years of age with a reference amount ≥200 g; (iv) 10% DV for foods for children 1–4 years of age with a reference amount ≤30 g or 30 mL; (v) 15% DV for foods for children 1–4 years of age with a reference amount >30 g or 30 mL; and (vi) 30% DV for foods for children 1–4 years of age with a reference amount ≥170 g.

**Table 1:** Nutrient thresholds which would determine the display of a front-of-pack symbol according to Canadian front-of-pack labelling regulations.

Age groups	Reference amount	Thresholds, %DV	Thresholds, absolute amount per nutrient		
			Saturated fat (g)	Sodium (mg)	Sugars (g)
Adults and children >4 years of age	>30 g or 30 mL	10%	2	230	10
	≤30 g or 30 mL	15%	3	350^a^	15
	≥200 g	30%	6	690	30
Children 1–4 years of age	>30 g or 30 mL	10%	1	120	5
	≤30 g or 30 mL	15%	1.5	180	8^a^
	≥170 g	30%	3	360	15

^a^The values are adjusted according to the rounding rules for nutrition labelling information as per *Food and Drug Regulations* [37]. Abbreviations: %DV, Percent Daily Value.

Foods falling into one of three exemption criteria would not display a FOP symbol regardless of their nutrient levels. The first exemption is health-related, where foods that have shown to have a recognized health protection benefit would be exempted from the regulations, including milk, eggs, fruits and vegetables. Additional exemption conditions have been set to identify foods that are important sources of calcium, as there is a high prevalence of inadequate intakes among Canadians [38]. Cheese and yogurt products high in calcium content (defined as ≥10% DV per reference amount for products with a reference amount ≤30 g or 30 mL; and ≥15%DV per serving size for products with a reference amount >30 g or 30 mL) would be exempted from displaying a ‘high-in’ FOP nutrition symbol, regardless of their levels of saturated fat and sodium. Second, technical exemptions are granted for foods that are already exempted from carrying NFt, which includes fresh fruits and vegetables, single ingredient meats, foods sold in very small packages, and foods sold at farmers’ markets. Under this exemption criteria, FOP symbol exemptions for ground meats with no added ingredients, are also exempted as they have similar nutrient value as whole cut meats. Third, practical exemptions are granted for foods that are known sources of the target nutrients (e.g., honey, syrup, salt, butter) as the FOP symbol may provide redundant information to consumers are exempted from the regulations.

All foods were categorized based on the total number of ‘high in’ nutrient(s) (i.e., exempted from regulations, no FOP symbol, a FOP symbol for 1 nutrient, a FOP symbol for 2 nutrients, and a FOP symbol for 3 nutrients) and the type of ‘high in’ nutrient (i.e., saturated fat, sodium, and sugars) that a food would display, similar to the categories used in the nutrient profiling model developed using the pre-published FOP labelling regulations [39].

### Dietary intake data

First-day 24-hour recall data from CCHS were matched to the CNF database that was classified using the Canadian FOP labelling regulations nutrient profiling model. Intakes of energy and nutrients-of-concern from foods categorized based on (1) the total number of ‘high in’ nutrients and (2) the type of ‘high in’ nutrient were summed for each individual.

Although free sugars, rather than total sugars, are one of the nutrients-of-concern for Canadians with a national dietary recommendation to limit consumption to <10% of energy from free sugars per total energy a day [40], Canadian FOP labelling regulations set thresholds for total sugars, as they are one of the mandatory nutrients presented on the NFt with %DV [41]. Therefore, in addition to intakes of total sugars, free sugar intakes and the top food categories contributing to free sugar intakes according to Canadian FOP labelling regulations were examined. The free sugar levels for foods in CNF were estimated using the 10-step added sugar decision algorithm by Wang et al. [42].

There were 357 foods (~5% of total available in CNF) that had missing levels of saturated fat, sodium, and/or total sugars; unless these foods were exempted from the FOP labelling regulations [15], they were considered to have levels below the threshold levels (i.e., not display a FOP symbol). Foods away from home, defined as foods consumed in a limited-service or full-service restaurants [43], were excluded from the analysis, as FOP labelling regulations do not apply to these foods.

### Statistical analysis

All statistical analyses were performed using SAS version 9.4, (SAS Institute Inc., Cary, NC, USA). The balanced repeated replication technique with 500 replicates using bootstrap weights and sample survey weights provided by Statistics Canada were applied to obtain accurate measures of variance and representative population-level estimates, respectively, appropriate for the CCHS survey design. Least square means and 95% CI were calculated using PROC SURVEYREG, adjusted for potential confounders in the model (age, sex, BMI, energy intake [for sodium only as intakes for other nutrients are expressed as a proportion to total energy intake], and misreporting status [i.e., under-, plausible-, and over-reporters)] for energy and nutrient intakes from foods according to Canadian FOP labelling regulations.

Top food categories contributing to energy and nutrient-of-concern intakes from foods that would be exempted from the FOP labelling regulations, display no FOP symbol (i.e., below threshold levels), and display a FOP symbol (i.e., ≥ threshold levels) were ranked using the population ratio method. The population ratio method was used to estimate the intake at the population level by summing the nutrient intakes by each food category from all individuals then divided by the total nutrient intake from all food categories from all individuals [44].

## Results

### Respondent characteristics

**Table 2** shows the characteristics of respondents included in the analysis. A total of 13,495 respondents were included in the analysis with a mean age [95% CI] of 49.3 years [48.8, 49.7], 52.8% of respondents were females, and 35.9% had a household income greater than $80,000/year. More than 83.2% of the respondents reported having at least a high school diploma or equivalency certificate. Based on the measured or adjusted BMI, 28.4% of respondents were normal-weight, 32.4% had overweight and 27.0% had obesity.

**Table 2:** Participant characteristics.

Characteristics	n (%) or means [95%CI]
**Females**, n (%)	7,124 (52.8)
**Age,** means [95% CI]	49.3 [48.8, 49.7]
**Total Annual Household Income**, n (%)	
< $20,000	1,510 (11.2)
$20,000 –$39,999	2,840 (21.0)
$40,000 –$59,999	2,418 (17.9)
$60,000 –$79,999	1,882 (13.9)
$80,000 –$99,999	1,350 (10.0)
$100,000 –$119,999	1,115 (8.3)
$120,000 –$139,999	706 (5.2)
> $140,000	1,667 (12.4)
Undisclosed	7 (0.1)
**Highest Level of Education**, n (%)	
Less than high school diploma or equivalency certificate	2,179 (16.1)
High school diploma or equivalency certificate	3,509 (26.0)
College, CEGEP, or other university certificate	4,488 (33.3)
Bachelor’s degree or university certificate above Bachelor’s degree	3,230 (23.9)
Undisclosed	89 (0.7)
**BMI**^**a**^, n (%)	
Normal weight (18.5–24.9 kg/m^2^)	3,832 (28.4)
Overweight (25.0–29.9 kg/m^2^)	4,367 (32.4)
Obese (≥30.0 kg/m^2^)	3,639 (27.0)
Undisclosed	1,657 (12.3)
**Misreporter**^**b**^, n (%)	
Under-reporter	5,912 (43.8)
Plausible	6,802 (50.4)
Over-reporter	781 (5.8)

Values are presented as weighted frequency, n (%) or means [95% CI]. n = 13,495. ^a^Self-reported Body Mass Index (BMI) was adjusted using the adjustment factor provided by Statistics Canada [33]. ^b^Misreporters were defined as respondents with the ratio of reported Energy Intake (EI) to Total Energy Expenditure (TEE; estimated using the Institute of Medicine equations [32]) *<*0.7 (under-reporters) and *>*1.42 (over-reporters).

### Energy and nutrient intakes

**Fig 1:**
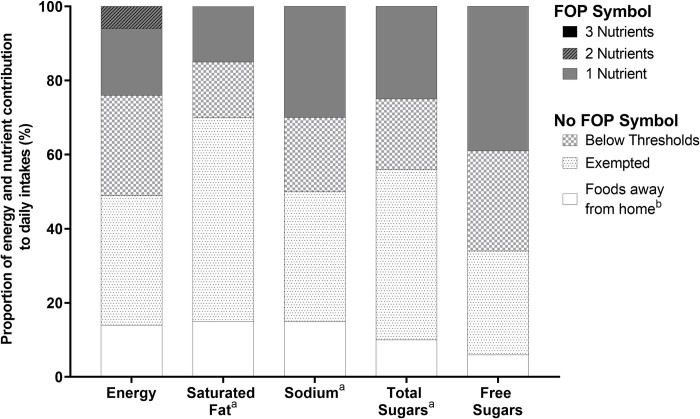
Proportion of energy and nutrient contribution consumed by Canadian adults according to Canadian front-of-pack (FOP) labelling regulations categories. Intakes of energy and nutrients-of-concern from foods were examined using the Canadian Community Health Survey-Nutrition 2015 (n = 13,495). ^a^Indicates nutrients that will be highlighted using a ‘high in’ front-of-pack (FOP) nutrition symbol according to Canadian FOP labelling regulations. ^b^Foods away from home were defined as foods consumed in a limited-service or full-service restaurant, that are excluded from Canadian FOP labelling regulations.

**Fig 1** shows the summary of energy and nutrient intakes from foods categorized according to FOP labelling regulations and foods away from home (excluded from the analysis). On average, 62% of consumed foods (1,378 kcal/d [95%CI: 1,329, 1,427] would not display a FOP symbol, while 24% of foods (534 kcal/d [504, 563]) would display a FOP symbol. Specifically, 35% (765 kcal/d [735, 795]) of total energy intake came from foods that would be exempted from FOP labelling regulations, while 27% (618 kcal/d [563, 673]) came from foods that were below threshold levels for all 3 target nutrients-of-concern. The majority of the consumed foods that would display a FOP symbol would have only 1 ‘high in’ nutrient (i.e., exceeding one of 3 target nutrients-of-concern), accounting for 18% (396 kcal/d [375, 417]) of intakes, and foods that would display a FOP symbol for 2 nutrients and for 3 nutrients accounted for 6% (136 kcal/d [123, 149]) and 0.1% (2 kcal/d [1, 4]), respectively.

On average, 55% (6.0% of total energy intake [5.8, 6.2]) of saturated fat intakes came from pre-packaged foods that would be exempted from FOP labelling regulations, 15% (1.3% of total energy intake [1.1, 1.6]) from foods that would not display a FOP symbol, as levels were below the saturated fat threshold levels, and 16% (1.9% of total energy intake [1.8, 2.0]) from foods that would display a FOP symbol for saturated fat. For sodium intakes, 35% (911 mg/d [834, 989]) of consumed foods would be exempted from FOP labelling regulations, 20% (473 mg/d [436, 509]) from foods that would not display a FOP symbol for being below the sodium threshold levels, and 30% (874 mg/d [811, 937]) from foods that would display a FOP symbol for sodium. For total sugar intakes, 46% (8.7% of total energy intake [8.2, 9.2]) came from foods that would be exempted from FOP labelling regulations, 19% (3.2% [2.7, 3.8] of total energy intake) from foods that would not display a FOP symbol for being below the sugar threshold levels, and 25% (5.7% of total energy intake [4.9, 6.5]) from foods that would display a FOP symbol for sugar. Canadians consumed approximately 28% (2.8% of total energy intake [2.5, 3.1]) of free sugar intakes from foods that would be exempted from FOP labelling regulations, 27% (1.7% of total energy intake [1.3, 2.0]) from foods below the sugar threshold levels, and 39% (5.0% of total energy intake [4.7, 5.3]) from foods that would display a FOP symbol for sugar.

Foods away from home contributed to approximately 14% of total energy (339 kcal/d [291, 388], 15% of saturated fat (1.6% of total energy intake [1.2, 2.0]), 15% of sodium (484 mg/d [404, 563]), 10% of total sugar (1.8% of total energy intake [1.3, 2.3]), and 6% of free sugar (0.8% of total energy intake [0.1, 1.5]) intakes.

### Top food categories contributing to energy and nutrient intakes

**Table 3** shows the summary of the top 3 food categories contributing to energy and nutrient-of concern intakes from foods that would be exempted from the FOP labelling regulations, that would not display a FOP symbol (i.e., below threshold levels), and that would display a FOP symbol for 1–3 nutrients-of-concern. The top 3 food categories contributing to energy and nutrient-of-concern intakes from foods that would be exempted from the FOP labelling regulations accounted for 14–40% of intakes, the top 3 food categories that would not display a FOP symbol due to below threshold levels accounted for 5–10% of intakes, and the top 3 food categories that would display a FOP symbol accounted for 10–43% of intakes.

**Table 3:** Top food categories contributing to energy and nutrient-of-concern intakes, according to Canadian front-of-pack labelling regulations.

	Rank	Exempted from regulations (average proportion in %)	No FOP symbol for the select nutrient-of-concern (average proportion in %)	FOP symbol for the select nutrient-of-concern (average proportion in %)
Energy	1	Fresh, frozen meats and substitutes (6.0)	Cheese, processed cheese, and cheese substitutes (3.3)	Breads (6.6)
	2	Canned, fresh, frozen fruits (4.5)	Breads (3.1)	Processed meat and meat substitutes (2.1)
	3	Butter, margarine, other fat (4.4)	Flour (2.9)	Fruit juices and drinks (1.9)
Saturated Fat^a^	1	Butter, margarine, other fat (13.2)	Breads (2.4)	Processed meat and meat substitutes (4.7)
	2	Cheese, processed cheese, and cheese substitutes (12.5)	Nut butter (1.3)	Chocolate and candies (4.2)
	3	Fresh, frozen meats and substitutes (6.7)	Mayonnaise and dressing (1.2)	Frozen desserts (4.1)
Sodium^a^	1	Salt and substitutes (16.6)	Breads (3.2)	Breads (10.1)
	2	Cheese, processed cheese, and cheese substitutes (5.2)	Condiments (1.6)	Processed meat and substitutes (8.1)
	3	Milk and substitutes (2.3)	Carbonated and non-carbonated beverages (1.3)	Soups (6.0)
Total Sugars^a^	1	Canned, fresh, and frozen fruits (15.8)	Breads (3.8)	Fruit juices and drinks (11.4)
	2	Sugars and substitutes (10.7)	Cakes, muffins, cookies, and other baked goods (1.2)	Carbonated and non-carbonated beverages (10.2)
	3	Milk and substitutes (8.2)	Honey, jam, bread spreads (1.1)	Chocolate and candies (5.0)
Free Sugars	1	Sugars and substitutes (20.4)	Breads (3.2)	Fruit juices and drinks (17.6)
	2	Honey, jam, bread spreads (4.7)	Honey, jam, bread spreads (2.0)	Carbonated and non-carbonated beverages (17.0)
	3	Canned, fresh, and frozen fruits (0.6)	Cakes, muffins, cookies, and other baked goods (2.0)	Chocolate and candies (8.0)

^a^Indicates nutrients that will be highlighted using a ‘high in’ front-of-pack (FOP) nutrition symbol according to Canadian FOP labelling regulations [15]. The top food categories are based on the average proportion (%) of each dietary component contributed, calculated using the population ratio method [44]. n = 13,495. Abbreviations: FOP, Front-of-pack.

The Processed meat and meat substitutes category was the top source for saturated fat intake from foods that would display a FOP symbol for saturated fat (4.7%). The Breads category was the top source for energy and sodium intake of foods that would display a FOP symbol for exceeding thresholds (6.6% and 10.1%, respectively). The Fruit juices and drinks category was the top source for total and free sugar intake from foods that would display a FOP symbol for sugars (11.4% and 17.6%, respectively).

## Discussion

The objective of the present study assessed the intakes of nutrients-of-concern from foods that would display a FOP symbol and the top food categories contributing to their intakes among Canadian adults. About a quarter of energy consumed by Canadians reported in CCHS 2015 came from foods that would display a FOP symbol for meeting or exceeding threshold levels for 1–3 nutrients-of-concern. The top food categories contributing to intakes of each nutrient-of-concern were nutrient-specific with Processed meat and meat substitutes as the top food category contributing to intakes for saturated fat, Breads for sodium, and Fruit juices and drinks for total and free sugars.

We found Canadians consumed approximately a quarter of their total energy from “less healthy” foods (i.e., foods that would display a FOP symbol) and these foods accounted for up to 40% of intakes of nutrients-of-concern. Consistent with previous studies showing a high prevalence of pre-packaged foods that are high in levels of nutrients-of-concern in Canada [39, 45, 46] and a high consumption of pre-packaged foods among Canadians [47, 48], our findings highlight the challenges Canadians currently face in identifying foods ‘high in’ nutrients-of-concern. Canada mandates the standardized back-of-pack nutrition labelling in the form of the NFt, which includes the mandatory declaration of energy, macronutrients, and some micronutrients per serving size [37]. Although more than half of Canadians report using NFt to make purchasing decisions [49], interpretive and easy-to-use FOP symbols and labels have been shown to help consumers more easily and accurately identify the healthfulness of foods compared to the NFt alone [50–52], particularly for those with low nutrition literacy skills [53, 54]. FOP labels can help individuals of various health literacy levels quickly and easily identify foods ‘high in’ nutrients-of-concern to make informed purchasing and consumption decisions, which can ultimately alter the intakes of these nutrients. Future studies assessing food choices and dietary intakes after the implementation of FOP labelling regulations and comparing them to the current findings will be needed.

Although FOP labelling regulations have the potential to help consumers prioritize nutrients-of-concern in pre-packaged foods, the extent of the impact of the regulations on the food supply, nutrient intakes, and health outcomes need to be monitored over time. For instance, FOP labelling regulations in Chile led to a decrease in the availability of foods that displayed ‘high-in’ FOP labels [14] and household purchasing of sugar-sweetened beverages [13]. Chile’s FOP labelling regulations established threshold levels for energy and 3 nutrients-of-concern (i.e., saturated fat, sodium, and sugars) that are generally more stringent than Canadian thresholds with a ‘stop-sign’ FOP label for each nutrient, which are more associated with a ‘warning’ message and take up more visual package space than the Canadian FOP symbol [55]. The Chilean FOP labelling regulations were also tied to restrictions on marketing to children and availability in schools of foods that would display FOP label(s) [55]. Considering the high prevalence of ‘less healthy’ foods marketed to children [56–58] and the high prevalence of highly processed foods in Canada [59], additional regulations, such as mandatory restrictions on marketing to children, and complementary public health and nutrition education [4, 7], similar to those introduced in Chile, may be needed to support a healthy food environment. As recommended by the World Health Organization [7], periodic monitoring and evaluation measures for FOP labelling regulations at the country level are needed to assess policy outcomes and address any policy gaps.

The top food categories contributing to intakes of nutrients from foods that would display a FOP symbol are consistent with foods-to-limit in the Canadian dietary guidelines, highlighting the potential impact that FOP labelling regulations can have on improving dietary patterns. Consistent with the WHO recommendation on FOP labelling systems to use a system that categorizes foods similarly to other national dietary guidelines and standards [7], our findings suggest that the Canadian FOP labelling regulations would similarly affect the food categories to limit (e.g., processed meats, fruit juices, sugar-sweetened beverages) according to Canada’s Food Guide (the national dietary guidelines for Canadians [60]) and other healthy dietary patterns (e.g., Dietary Approaches to Stop Hypertension [DASH] [61]). Product reformulations may help decrease the overall intake of nutrients-of-concern from these food categories. However, an earlier analysis of the Canadian food supply using the pre-published *Canada Gazette I* [62], proposals for FOP labelling revealed a high prevalence of food products in these categories that would display a FOP symbol [39]. For instance, ≥90% of products in Fruit juices and drinks and Meat and substitutes categories would have needed to display a FOP symbol as of 2017 [39], suggesting a potential need for other public health measures to promote dietary substitutions across food categories rather than within food categories (e.g., Nuts & Seeds for Processed meats). Within food category substitutions may be more suitable for other commonly-consumed food categories that can have a more diverse nutritional profile of foods (e.g., Breads), given positive industry reformulation responses take place to improve the availability of ‘more healthy’ foods in these categories. As of 2017, over 60% of foods in the Bakery Products category (which includes breads) available in the Canadian food supply would have needed to display a FOP symbol [39]; but, to date, there has been a poor track record in responding to voluntary initiatives to reduce sodium levels [63]. In addition to other public health strategies to promote food substitutions for healthy dietary behaviours, periodic evaluation of the food supply will be needed to evaluate the effectiveness of the regulations.

Unfortunately, a significant portion of intakes came from foods that are exempted from FOP labelling regulations, which may blunt the potential impact of FOP labelling regulations on the nutritional intakes of Canadians. First, the exemption criteria of foods of ‘well-known sources’ of target nutrients [15] contributed to 13–20% of intakes of nutrients-of-concern. A previous study showed that despite the frequent consumption of foods prepared at home by Canadians, dietary quality of foods prepared at home was not necessarily “healthy,” with high proportional contribution to intakes of nutrients-of concern [64]. The lack of FOP symbol on culinary ingredients may lead to consumer confusion around their health benefits (e.g., white sugar vs. molasses, butter vs. oil, table salt vs. kosher salt), which could prevent consumers from reducing the use and/or substituting less healthy culinary ingredients for healthier alternatives (e.g., herbs for salt, oils high in unsaturated fat for butter). Second, Canadian FOP labelling regulations published in 2022 had a number of changes from the pre-publication of the regulations in 2018 [62]. Although the target nutrients-of-concern (i.e., saturated fat, sodium, total sugars) remained the same, several aspects of the final FOP labelling regulations were changed. The most notable change was the exemptions for some dairy products (i.e., cheese and yogurt products with high calcium content [15]), which will be subjected to re-evaluation in 10 years [15]. With a high proportion of foods exempted from FOP labelling regulations, the effectiveness of the exemption criteria must be part of the policy evaluation plan to help consumers make informed dietary choices.

Consistent with previous findings, a significant portion of energy and nutrients-of-concern came from restaurant foods and other foods consumed away from home [43, 64], which are not included in FOP labelling regulations. A previous study showed individuals consuming restaurant foods had overall lower diet quality with higher intakes of nutrients-of-public health concern compared with individuals who did not consume any restaurant foods [43]. Sodium is of particular concern for restaurant foods, where they contributed to over 20% of daily intakes in the current study. A recent study examining the nutrition information of foods from over 200 chain restaurants in Canada revealed high sodium levels in restaurant foods, with an average of 1,588 mg per serving among starters (69% of the CDRR level of 2,300 mg/d) and 1,232 mg per serving among entrées (54% of the CDRR level) [65]. Although most FOP labelling regulations and voluntary systems around the world (including Canada) only apply to pre-packaged foods [3, 6, 66], it could be used in other settings, including restaurants [67, 68]. Further investigation on public health policies that can improve the quality of foods consumed away from home and consumer behaviors are needed to further improve the dietary intakes of a population.

There are a few methodological limitations to consider. First, we focused on the intakes of energy and nutrients-of-concern to assess the potential implications of the Canadian FOP labelling regulations; therefore, we did not examine other aspects of the respondents’ diet. Although energy-dense and nutrient-poor diets tend to be associated with poor diet quality, overall diet measures, which can include food groups, nutrients-of-concern, and nutrients-to-encourage, have shown to be more strongly associated with disease risk than single-nutrient approaches [69, 70]. In addition to monitoring nutrient intakes to evaluate the regulations, dietary quality should also be monitored to ensure that dietary patterns associated with lower health risk are adapted by Canadians as intakes of nutrients-of-concern decrease. Second, the present analysis was conducted using single-day dietary data. Although within-person, day-to-day variation can affect single-day dietary data [71], the current study aimed to assess average population intakes; therefore, suitable for this study design [72]. Lastly, the CCHS dietary data is linked to CNF, a generic food composition database, which was last updated in 2015 for the CCHS 2015 [35]. CNF may not accurately reflect the current nutrient content of foods in the Canadian food supply. A branded nutrient database, Food Label Information and Price (FLIP), has been shown to better reflect the foods that would be affected by FOP labelling regulations than the generic food composition database [39], since it shows the average nutrient levels of similar pre-packaged foods available in the market [35]. The use of branded food composition databases (e.g., FLIP) will be needed to accurately monitor the impact of FOP labelling regulations on dietary intakes.

## Conclusions

Canadian FOP labelling regulations have the potential to influence the intakes of nutrients-of-concern for Canadians. However, to achieve the objectives of the regulations, complementary public health strategies, ongoing monitoring of the outcomes, and potential revisions to the current exemption criteria may be needed. FOP labelling regulations can be an effective public health strategy to improve dietary intakes and address the rising prevalence of NCDs in Canada and around the world. As mandatory FOP labelling regulations are implemented globally, monitoring and evaluation are needed to identify and address any potential policy gaps.

## Supporting information

S1 Table Summary of food categories. A food category list of 62 used to identify top food categories contributing to intakes of energy and nutrients-of-concern consumed by Canadian adults.
(DOCX)
