# Correction: Nutrient intakes and top food categories contributing to intakes of energy and nutrients-of-concern consumed by Canadian adults that would require a ‘high-in’ front-of-pack symbol according to Canadian labelling regulations

**DOI:** 10.1371/journal.pone.0306212

**Published:** 2024-06-24

**Authors:** Jennifer J. Lee, Mavra Ahmed, Alena (Praneet) Ng, Christine Mulligan, Nadia Flexner, Mary R. L’Abbé

In Table 1, the values in the “reference amount” column are incorrect. Please see the correct Table 1 here.

**Table 1:** Nutrient thresholds which would determine the display of a front-of-pack symbol according to Canadian front-of-pack labelling regulations.

Age groups	Reference amount	Thresholds, %DV	Thresholds, absolute amount per nutrient		
			Saturated fat (g)	Sodium (mg)	Sugars (g)
Adults and children >4 years of age	≤30 g or 30 mL	10%	2	230	10
	>30 g or 30 mL and <200 g	15%	3	350^a^	15
	≥200 g	30%	6	690	30
Children 1-4 years of age	≤30 g or 30 mL	10%	1	120	5
	>30 g or 30 mL and <170 g	15%	1.5	180	8^a^
	≥170 g	30%	3	360	15

^a^The values are adjusted according to the rounding rules for nutrition labelling information as per *Food and Drug Regulations [37]*. Abbreviations: %DV, Percent Daily Value.
