# Supplementary material for: Nutrient intakes and top food categories contributing to intakes of energy and nutrients-of-concern consumed by Canadian adults that would require a ‘high-in’ front-of-pack symbol according to Canadian labelling regulations
Source: PLoS One. 2023 May 18;18(5):e0285095. doi: 10.1371/journal.pone.0285095 (PMC10194982; doi:10.1371/journal.pone.0285095)
Supplement: S1 Table — A food category list of 62 used to identify top food categories contributing to intakes of energy and nutrients-of-concern consumed by Canadian adults. (DOCX) [file pone.0285095.s001.docx]

**Table of Contents**

[**S1 Table.** Summary of Food Categories 2](#_Toc122346716)

# **S1 Table.** Summary of Food Categories

| **Name** | **Examples** | **Matching TRA Minor Categories^a^** |
| --- | --- | --- |
| **Bakery Products** |  |  |
| Breads | Breads (including quick type), tea biscuits, scones, rolls, buns, English muffins, croissants, tortillas, pitas, soft breads, bagels, naans, flat bread, pizza crust, taco shell | A.1, A.2, A.3, A.24, A.25, A.26 |
| Cakes, cookies, and other baked goods | Brownies, cakes, coffee cakes, donuts, Danishes, sweet rolls | A.4, A.5, A.6, A.7, A.8, A.9, A.10, A.14, A.21, A.22, A.23 |
| Crackers | Accompaniment crackers, hard bread sticks, melba toast, snack crackers, dry breads, rusks | A.11, A.12, A.13 |
| Pancakes and waffles | French toast, pancakes, waffles | A.17 |
| Granola and energy bars | Grain-based bars, energy bars, protein bars | A.18, A.19, A.20 |
| **Cereals and Other Grain Products** | | |
| Hot cereals | Oatmeal, cream of wheat | C.1 |
| Ready-to-eat cereals | Puffed and coated/uncoated | C.2, C.3 |
| Granola cereals | Fruit and nut included cereals, biscuit type cereals | C.4 |
| Flour | Bran and wheat germ, flours (including wheat, potato, barley, nut), cornmeal | C.5, C.6, O.4 |
| Grains | Rice, barley | C.7 |
| Pastas | Pastas | C.8, C.9 |
| **Dairy & Dairy Substitutes** |  |  |
| Cheese, processed cheese, and cheese substitutes | Cream cheese, cheese spread, flavoured cheese, shredded cheese, cottage cheese, ricotta cheese, quarks, romano cheese, cheddar cheese | D.1, D.2, D.3, D.4, D.5 |
| Evaporated milk, cream, and cream substitutes | Cream, milk & cream blend, sour cream, evaporated/condensed milk, coconut milk | D.6, D.7, D.8, D.14, D10, M.11 |
| Yogurt | Yogurt in tubs, drinkable yogurt, kefir | D.12, D.15 |
| **Sweets & Desserts** |  |  |
| Frozen desserts | Ice cream, ice milk, frozen yogurt, sherbet, ice cream cone | E.1, E.2, E.3, E.4, A.15 |
| Custard and pudding | Custard, gelatin, pudding | E.5, E.16 |
| Chocolate and candies | Confectionaries, chocolate, candies, gum, mints, gummies, marshmallow, halva | M.7, U.1, U.2, U.3, U.4, U.5, U.6, U.10, U.11, O.2 |
| **Egg & Egg Substitutes** |  |  |
| Egg and substitutes | Fresh and pre-packaged eggs, pickled eggs, quail eggs, egg mixtures, liquid egg whites, egg powder | G.1, G.2, G.3, G.4 |
| **Meat, Poultry, & Meat Substitutes** | | |
| Bacon and substitutes | Bacon, bacon-like beef, pork, poultry strips | L.1, L.2 |
| Processed meat and substitutes | Dried meat, luncheon meat, sausages, cured meat, canned meat | L.3, L.4, L.5, L.8, L.9, L.11 |
| Fresh, frozen meats and substitutes | Fresh, frozen meats with and without sauce | L.6, L.10 |
| Patties and other meat substitutes | Burger patties, cutlets, meatballs, sausage meats, ground meat, falafels, simulated meat products | L.7, K.1 |
| **Fats & Oils** |  |  |
| Butter, margarine, other fat | Butter, margarine, shortening, lard, solid coconut oil | H.1, H.3 |
| Oil | Vegetable oil, spray oil | H.2, H.6 |
| Mayonnaise and Dressing | Mayonnaise, sandwich spread, other salad dressings | H.4, H.5 |
| **Seafood & Substitutes** |  |  |
| Fresh/frozen seafood and substitutes | Raw, cooked with and without sauce | I.2, I.3 |
| Canned and pickled seafood | Anchovies, caviar, canned tuna, smoked salmon, pickled herrings | I.1, I.4, I.5 |
| **Fruits** |  |  |
| Canned, fresh, and frozen fruits | All fruits (including avocados), lemon/lime juices used as ingredients | J.1, J.2, J.3, J.4, J.5, J.12 |
| Prepared fruits | Applesauce, dried fruit, pickled fruit, fruit for garnish, fruit relishes | J.6, J.7, J.8, J.9, J.10, J.13, J.14, M.12 |
| **Vegetables** |  |  |
| Fresh, frozen, canned vegetables | Fresh, frozen, canned non-starchy vegetables with and without sauce | V.1, V.2, V.3, V.4, V.5, V.6 |
| Fresh, frozen potatoes and starchy vegetables | Fresh, frozen, mashed potatoes | P.1, P.2, P.3 |
| Pickled vegetables | Olives, sun-dried tomatoes, pickled vegetables, relish | V.8, V.9, V.10 |
| **Legumes & Pulses** |  |  |
| Legumes | Dry, canned legumes | K.2 |
| Prepared legumes | Refried beans, dried texturized soy protein and isolate | K.3, K.4 |
| **Mixed Dishes** |  |  |
| Mixed entrees | Burritos, chicken and rice casserole, mac and cheese, lasagna, stir fry | N.1, N.2 |
| Appetizers | Hors d'oeuvres | N.3 |
| Soups | Canned, dry, frozen, pre-made soups | T.1 |
| Salads | Vegetable, eggs, fish, bean, gelatin, pasta, potato | Q.1, Q.2, Q.3 |
| **Sauces and Condiments** |  |  |
| Dips and dipping sauce | Sweet and sour sauce, plum sauce, cocktail sauce, vegetable and legume dips | R.1, R.2 |
| Entrée sauce | Spaghetti sauce, butter chicken, pad Thai, chicken wing sauce, pesto, tomato paste, tomato sauce, gravy | R.3, R.4, V.11, V.12 |
| Condiments | Ketchup, mustard, chili sauce, soy sauce, vinegar, hot sauce, Worcestershire, sauce | R.5, R.6 R.7 |
| **Snacks** |  |  |
| Potato chips, popcorn, pretzels, and other chips | Potato chips, corn chips, rice chips, pretzels, pulse-based chips, fruit chips, pita chips, seaweed snacks | S.1, S.3 |
| Nuts and seeds | Coated and uncoated, mixes with chocolate and dried fruit | S.2, O.1 |
| **Beverages** |  |  |
| Carbonated and non-carbonated beverages | Sugar-sweetened beverages, artificially sweetened beverages, energy drinks, vitamin water, coconut water, cocoa and chocolate beverages, non-alcoholic beverages | B.1, B.5 |
| Alcoholic beverages | Alcoholic beverages | B.2 |
| Coffee & Tea | Sweetened and unsweetened coffee & tea | B.3, B.4 |
| Eggnog | Eggnog | D.9 |
| Milk and substitutes | Milk (all fat levels), plant-based dairy substitutes (inc. Almonds, cashew, soy, coconut) | D.11 |
| Shakes | Dairy or dairy-alternative based shakes | D.13 |
| Fruit juices and drinks | Fruit juices, nectars, fruit drinks | J.11 |
| Vegetable juices and drinks | Vegetable juices, drinks, cocktails | V.7 |
| **Miscellaneous** |  |  |
| Baking ingredients | Baking powder, baking soda, yeast, starch, cocoa powder, carob powder | M.1, M.5, C.10 |
| Breadcrumbs and croutons | Breadcrumbs, batter mixes, croutons, stuffing, salad toppers | M.3, A.16, M.8, C.11 |
| Cooking wine | Cooking wine | M.4 |
| Salt and substitutes | Salt and salt-based seasonings | M.9 |
| Spices and herbs | Dry spices and herbs without salt | M.10 |
| Sweet baking ingredients & decorations | Maple butter, marshmallow cream, cake frostings, pie fillings, baking decorations | F.1, F.2, F3., M.2 |
| Sugars and substitutes | Sugars, artificial sweeteners | U.7, U.12, U.13 |
| Honey, jam, and bread spreads | Molasses, agave syrup, pancake syrups, maple syrups, high fructose corn syrup, flavoured syrups, Nutella | U.8, U.9, U.15, U.14, M.6 |
| Nut butter and substitutes | Peanut, almond butter | O.3 |

^a^Health Canada’s Table of References Amounts [1] were grouped to create consumer-friendly food category list of 62.

**REFERENCES**

1. Health Canada. Table of Reference Amounts for Food [Internet]. 2022 [cited November 24, 2022]. Available from: <https://www.canada.ca/en/health-canada/services/technical-documents-labelling-requirements/table-reference-amounts-food/nutrition-labelling.html>.
